# Impact of Eating Context on Dietary Choices of College Students: Evidence from the HEALTHY-UNICT Project

**DOI:** 10.3390/nu14204418

**Published:** 2022-10-21

**Authors:** Andrea Maugeri, Roberta Magnano San Lio, Giuliana Favara, Maria Clara La Rosa, Claudia La Mastra, Paolo Marco Riela, Luca Guarnera, Sebastiano Battiato, Martina Barchitta, Antonella Agodi

**Affiliations:** 1 Department of Medical and Surgical Sciences and Advanced Technologies “GF Ingrassia”, University of Catania, 95123 Catania, Italy; 2 Department of Mathematics and Informatics, University of Catania, 95123 Catania, Italy

**Keywords:** diet, dietary determinants, adolescence, meals, meal context, web-app, ecological momentary assessment

## Abstract

While personal characteristics have been evaluated as determinants of dietary choices over the years, only recently studies have looked at the impact of eating context. Examining eating context, however, can be challenging. Here, we propose the use of a web-app for the Ecological Momentary Assessment of dietary habits among 138 college students from Catania (Italy) and therefore for examining the impact of eating context on dietary choices. Eating away from home was associated with lower odds of consuming vegetables, fruits, and legumes and higher odds of consuming processed meat, salty snacks, and alcoholic drinks compared with eating at home. Eating in the company of other people was associated with higher odds of consuming vegetables, red meat, fish, legumes, milk, and sugar-sweetened beverages and lower odds of consuming nuts than eating alone. This study proposed a new way to capture and assess how eating environment might affect dietary habits. Based on our results, meal location and social context have significant effects on the dietary choices of college students, pointing to the need to incorporate these aspects into further epidemiological studies.

## 1. Introduction

Adolescence and adulthood are crucial periods for establishing healthy behaviors which will last a lifetime. However, this period often coincides with a deterioration in diet quality, a decline in physical activity, and a higher risk of overweight or obesity [1,2,3,4,5,6,7,8]. In line with the World Health Organization, indeed, health and well-being conditions of adolescent and young adults are related to lifestyle and behavioral factors, which make them more vulnerable to diseases and other adverse outcomes [9]. In particular, adolescents and young adults tend to have unhealthy eating habits, such as a low intake of fruit and vegetables and a high consumption of calorie-dense foods, which have both immediate and long-term physiological and pathological effects [10,11,12,13,14,15]. For this reason, identifying the main determinants associated with healthy food choices is imperative for the design of effective actions and interventions.

Although personal characteristics have been evaluated over the years as determinants of dietary choices [2,3,4,16], recent studies have focused on eating context, specifically the immediate environment in which individuals eat [17,18,19,20,21]. For example, previous research has examined the impact of eating context on diet quality in older adults, showing that dietary choices may be strongly influenced by where, with whom, and in what surroundings one eats [22]. Some studies have also evaluated the effect of eating context on the dietary consumption of children in specific settings, such as school. However, their approaches were designed to capture single meals and specific characteristics of the school environment [23,24]. Moreover, the identification of the main behavioral determinants among adolescent and young adults is still considered crucial for public health interventions, suggesting the need for innovative and more precise dietary assessment tools. In fact, examining eating context can often be challenging and has so far been accomplished via questionnaires assessing the characteristics of the eating environment. These methods, however, can only give some indications, but not a detailed assessment, of the context at the time of eating.

The Ecological Momentary Assessment (EMA) represents an innovative approach which can help overcome these limitations through real-time recording of multiple eating contexts as well as detailed dietary data on the same individual. As originally proposed, EMA was used to evaluate moods and emotions during real life events, but if applied to dietary assessment, it could reveal a great deal about dietary habits and physical activity, as well as where these activities take place. This then allows us to evaluate concurrent events and their strict relationships. This approach also has the potential to reduce recall biases and to investigate those factors influencing dietary habits [25]. A comprehensive review of methods and perspectives about the application of the EMA in nutritional epidemiology is given in a previous systematic review by Maugeri and Barchitta [25]. With these considerations in mind, the current study aimed to use a web-app for the EMA of dietary habits among college students. By examining where college students ate and their social context, the study also evaluated the impact of the eating context on their dietary choices.

## 2. Materials and Methods

### 2.1. Study Design and Popuation

The study was conducted as part of the HEALTHY-UNICT (Healthy diet and lifestyles among University of Catania Students: a mobile Ecological Momentary Assessment approach for health promotion) project, which recruited students from the University of Catania (Catania, Italy) to characterize their behaviors, anthropometric measures, and emotions. In this framework, as described elsewhere [26], we developed a web-app for the EMA of dietary consumption, physical activity, smoking habits, sleep duration and quality, and other daily activities. The web-app was designed and validated to collect dietary data in real time and in the real world [26]. The EMA employed in the HEALTHY-UNICT project was based on a previous systematic review [25] and consisted of a 7-day assessment with a signal-contingent approach [26]. The study population included a convenience sample of students from the hygiene and public health courses of the bachelor’s or master’s degree programs in the biological sciences, medicine, and the health professions at the University of Catania. This convenience sample was chosen because these students were probably more aware and motivated to use the web application. From April to June 2021, all students received information about the project and were invited by email to participate in the study. The study protocol was carried out in accordance with the Declaration of Helsinki and approved by the Ethics Committee Catania 1 with the following protocol number: n.70/2021/PO. All students were informed about the study protocol and signed an informed consent. In the current analysis, we used data from students who completed a one-week assessment during the second semester of the academic year 2020–2021. Students who did not respond to more than five notifications were excluded from the analysis.

### 2.2. Data Collection

At the time of recruitment, students were instructed to use the web-app and completed a registration questionnaire about sociodemographic characteristics, anthropometric measures, and usual behaviors. During the 7-day EMA assessment, students received by email five notifications per day, which prompted them to record their daily activities at predefined intervals (i.e., 7 a.m.–10 a.m., 10 a.m.–1 p.m., 1 p.m.–4 p.m., 4 p.m.–7 p.m., 7 p.m.–10 p.m.). In particular, dietary information was collected about foods and drinks consumed at mealtime, where the eating episode took place, and who was present during the eating event. Thus, for each eating occasion, data were available about meal type (breakfast, morning and afternoon snacks, lunch, or dinner), foods and drinks consumed (from a list of 38 food categories), meal location (at home, at work/university, public closed space, public open space), and social context (alone, with family, with friends, with colleagues). Next, modalities for meal location were categorized as “at home” versus “away from home” (i.e., including at work/university, public closed space, public open space); modalities for social context were categorized as “alone” versus “in company” (i.e., including with family, with friends, with colleagues).

### 2.3. Statistical Analysis

Descriptive statistics were used to characterize dietary data and information on eating context. Results are expressed as frequencies and percentages for categorical variables, or mean and standard deviation (SD) for continuous variables.

A Sunburst chart was used to depict the structure of hierarchical data as a set of nested rings: the inner ring referred to the meal type (breakfast, morning snack, lunch, afternoon snack, dinner); the medium ring referred to the meal location (at home versus away from home); and the outer ring referred to the social context (alone versus in company).

Logistic regression analyses were applied to identify features of the eating context that might be associated with dietary choices. The models included eating specific food categories as the dependent variable, while meal location (at home versus away from home) and social context (alone versus in company) were the independent variables. Each model was adjusted for age, gender, day of the week, meal type, and time of the day in which eating episodes occurred. An interaction term was included to evaluate the effect of gender on the observed relationships. Outputs of regression models were reported as odds ratios (ORs) and their 95% confidence intervals (95%CIs).

Employing clustering algorithms is another way to manage and analyze multivariate and multilevel data. For this reason, we applied the two-step clustering method to group eating episodes according to their main features (i.e., location, social context, day of the week). This method allows to handle categorical and continuous variables, using a likelihood distance measure that assumes that variables in the cluster model are independent. The procedure of the algorithm begins with construction of a cluster features tree, and then the nodes of the cluster features tree are grouped using an agglomerative clustering algorithm. To automatically determine the optimal number of clusters, each potential solution is compared using Schwarz’s Bayesian criterion (BIC).

All the analyses were conducted using the SPSS software (version 26.0, SPSS, Chicago, IL, USA), with a statistical significance level of 0.05.

## 3. Results

### 3.1. Population Characteristics

An invitation was sent to all 138 students enrolled in the hygiene and public health courses of the bachelor’s or master’s degree programs in the biological sciences, medicine, and the health professions at the University of Catania. Among them, 132 students (95.6%) with a mean age of 24.1 years (SD = 4.2 years; 75.5% females) responded to at least 30 notifications. In particular, 128 students (97%) answered all 35 notifications received during the one-week assessment. These students were enrolled in bachelor’s degrees (52.9%) or master’s degrees (47.1%) courses at the University of Catania. About half of them were non-resident students (49.3%), while the others were residents of Catania (23.9%) or commuters (26.8%). Most of them were non-smokers (79.7%), while the remaining were smokers (15.2%) or ex-smokers (5.1%). The average body mass index (BMI) was 22.9 kg/m^2^ (SD = 5.0 kg/m^2^), and 23.8% of students were overweight or obese.

### 3.2. Characteristics of Eating Episodes

**Figure 1:**
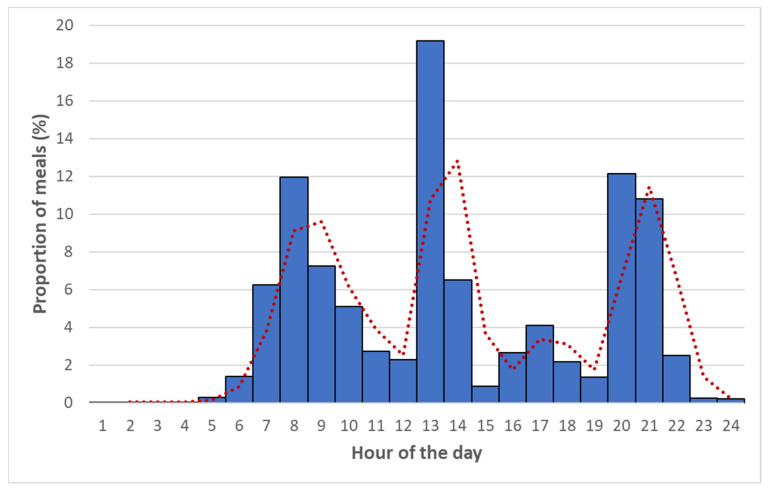
Proportion of meals consumed over the day. Bars represent the percentages of meals occurring at each hour of the day. Percentages are obtained by dividing the number of meals in each hour of the day by the total of all meals recorded. Red line represents the moving average line of the proportion of meals over the day.

**Figure 2:**
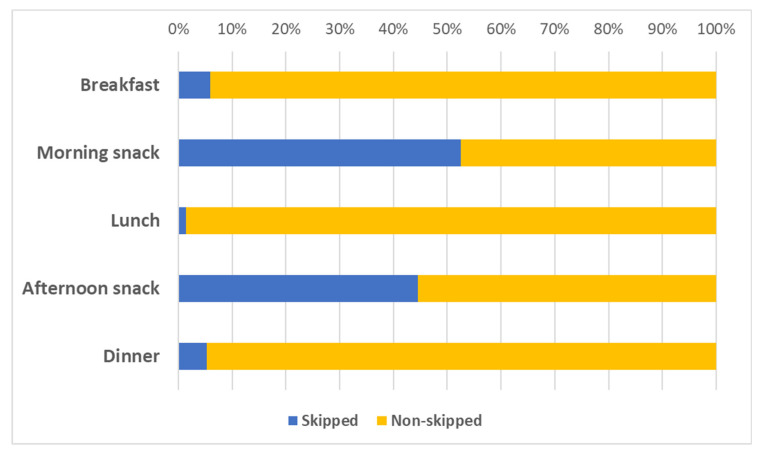
Proportion of skipped and non-skipped eating episodes for type of meal. Blue bars represent the proportion of skipped meals for each type of meal. Yellow bars represent the proportion of non-skipped meals for each type of meal.

For a total of 2770 data entries related to eating episodes, 466 meals (16.8%) were skipped. Overall, Figure 1 shows the proportion of eating episodes per time of the day, with three peaks coinciding with breakfast, lunch, and dinner. Accordingly, the proportion of skipped meals was 5.9% for breakfast, 52.5% for morning snack, 1.4% for lunch, 44.5% for afternoon snack, and 5.3% for dinner (Figure 2).

**Figure 3:**
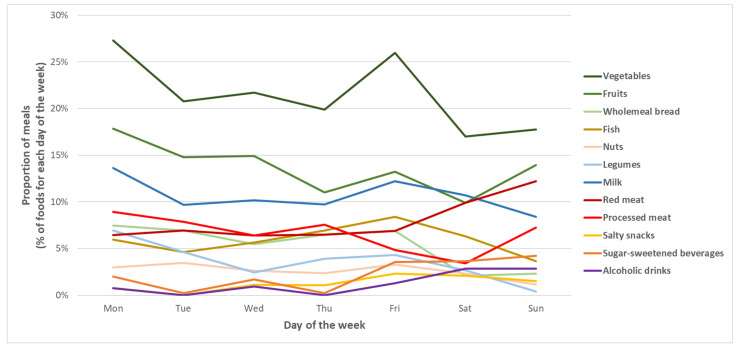
Proportions of meals over the week, stratified for specific foods. Lines represent trends of the percentages of meals occurring over the week for specific food categories. For each day of the week, proportions are obtained by dividing the number of meals including specific foods by the total number of meals.

In general, the proportions of foods consumed were almost constant over the week. However, an increase was evident for the consumption of red meat on the weekend as well as a decrease in the consumption of vegetables (*p*-values < 0.05; Figure 3). Most meals occurred at home (90.8%), followed by public open space (5.3%), public closed space (2.0%), and at work/university (1.9%). Moreover, 36.1% of meals occurred alone, while 55.7% occurred with family, 6.8% with friends, and 1.4% with colleagues. For this reason, from this point, meal location was categorized as at home versus away from home, while social context was categorized as alone versus with company.

**Figure 4:**
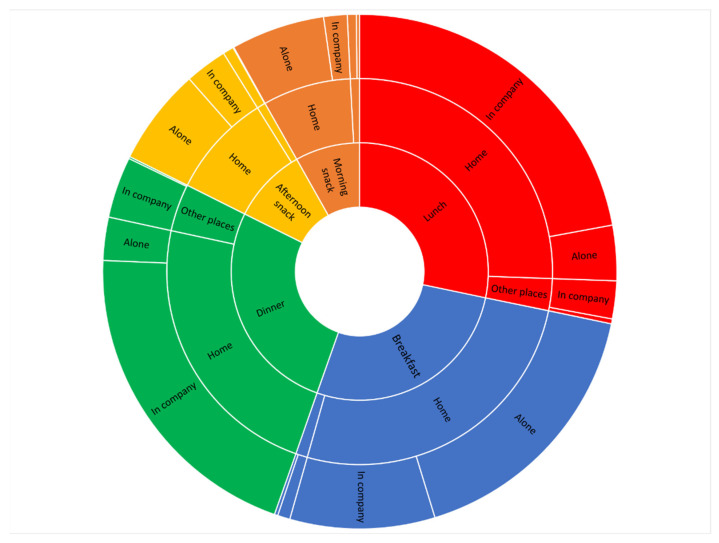
Sunburst chart of characteristics of eating episodes. This multilevel chart summarizes the distribution of eating episodes according to meal type, meal location, and social context. The size of each segment is proportional to the corresponding values. The inner ring refers to the meal type (breakfast, morning snack, lunch, afternoon snack, dinner); the medium ring refers to the meal location (at home versus other places); the outer ring refers to the social context (alone versus in company).

The Sunburst chart in Figure 4 summarizes the characteristics of eating episodes in a hierarchical way. This chart shows some differences in terms of meal location and social context by meal types. Specifically, the proportion of meals consumed at home was 96.3% for breakfast, 92.2% for afternoon snack, 90.4% for morning snack, 90.5% for lunch, and 85.2% for dinner. The proportion of meals consumed with company was 89.5% for dinner, 86.6% for lunch, 36.5% for breakfast, 35.3% for afternoon snack, and 25.0% for morning snack. By observing the chart, it is possible to visualize the proportions of eating episodes for each combination of meal type, meal location, and social context.

### 3.3. Association of Eating Context with Dietary Choices

To investigate the association between eating context and dietary choices, we performed logistic regression analyses, adjusting for age, gender, day of the week, meal type, and time of the day in which eating episodes occurred as covariates (Table 1). In particular, the multivariable analysis demonstrated that eating away from home was associated with lower odds of consuming vegetables (OR = 0.52; 95%CI = 0.36–0.76; *p* = 0.001), fruits (OR = 0.28; 95%CI = 0.16–0.51; *p* < 0.001), and legumes (OR = 0.13; 95%CI = 0.01–0.95; *p* = 0.044) and higher odds of consuming processed meat (OR = 1.81; 95%CI = 1.12–2.92; *p* = 0.015), salty snacks (OR = 4.25; 95%CI = 2.17–8.31; *p* < 0.001), and alcoholic drinks (OR = 18.42; 95%CI = 7.04–48.21; *p* < 0.001) than eating at home. Eating with company was associated with higher odds of consuming vegetables (OR = 3.09; 95%CI = 2.31–4.13; *p* < 0.001), red meat (OR = 4.39; 95%CI = 2.51–7.65; *p* < 0.001), fish (OR = 9.34; 95%CI = 4.22–20.65; *p* < 0.001), legumes (OR = 3.03; 95%CI = 1.56–5.88; *p* = 0.001), milk (OR = 1.51; 95%CI = 1.11–2.06; *p* = 0.009), and sugar-sweetened beverages (OR = 3.55; 95%CI = 1.58–7.99; *p* = 0.002) and lower odds of consuming nuts (OR = 0.27; 95%CI = 0.15–0.48; *p* < 0.001) than eating alone. No interaction with gender was evident in each regression model (*p*-values > 0.05 for the interaction).

### 3.4. Clusters of Eating Episodes

Cluster analysis identified four clusters of eating episodes according to their similarities and differences. The distributions of characteristics for each cluster are summarized in Table 2. Cluster 1 was characterized by eating episodes that mainly occurred away from home and with other people. This cluster was associated with a high consumption of red and processed meat, and sugar-sweetened beverages. Cluster 2 was characterized by eating episodes that occurred at home, with family, and in the workweek. This cluster was associated with high consumption of vegetables, fruit, legumes, and fish. Cluster 3 was characterized by eating episodes occurring alone, at home, and in the workweek. This cluster was associated with high consumption of nuts. Cluster 4 was characterized by eating episodes occurring at home, with family members, and on weekends. This cluster was associated with high consumption of red meat.

## 4. Discussion

In the framework of the “HEALTHY-UNICT” study, we examined the effect of eating context on the dietary choices of college students from Catania, Italy. In general, most of eating episodes occurred at home and in company with other people. In particular, social context and the location of each eating episode were associated with dietary choices of students included in our analysis. Meals occurring away from home were characterized by lower consumption of vegetables, fruits, and legumes and higher consumption of processed meat, salty snacks, and alcoholic drinks. Meals in the company of other people were characterized by higher consumption of vegetables, red meat, fish, legumes, milk, and sugar-sweetened beverages and lower consumption of nuts. These findings were also confirmed by applying alternative multivariate techniques for data analysis (i.e., cluster analysis).

To the best of our knowledge, only a few studies have incorporated all these aspects of eating episodes to identify the main determinants of dietary choices, especially among adolescents. Recently, Shams-White and colleagues analyzed the quality of diet of older adults—assessed through the Healthy Eating Index 2015 (HEI-2015)—according to different eating contexts [22]. The authors found lower diet quality for meals occurring away from home and with company. In fact, eating episodes occurring away from home were characterized by lower consumption of fruits, whole grains, and dairy. Instead, eating with other people was associated with higher consumption of vegetables and beans, and less consumption of added sugars [22]. These results were partially in line with ours and with a systematic review carried out in 2011 [27]. Lachat and colleagues, in fact, concluded that junk foods were the main types of foods consumed away from home by U.S. adults, leading to higher intake of calories and fats and lower intake of micronutrients [27]. An analysis of 10 European countries—in the framework of the European Prospective Investigation into Cancer and Nutrition study—confirmed the detrimental effect of eating away from home on dietary intakes. However, the authors also showed some differences by gender and between countries [28].

Moving to adolescents, a cross-sectional study in Vietnam examined the nutritional contribution of eating away from home and possible differences related to socioeconomic factors. The authors showed that eating away from home was associated with lower total energy intake, higher dietary diversity, and high consumption of fats and carbohydrates. In rural areas, however, eating away from home was associated with higher consumption of fruit, meat, poultry, and offal [29]. Another cross-sectional study, conducted on Albanian university students, reported a more frequent consumption of sweets, soft drinks, and meat products away from home, while the consumption of fruits and vegetables was extremely less frequent. Accordingly, meals occurring at home were characterized by higher intake of carbohydrates, total fats, and proteins, while those occurring away from home were richer in saturated fats [30].

The abovementioned studies, however, were mainly conducted administering a 24 h dietary recall, which is unsuitable for collecting information at the exact time of eating. In our study, we proposed a novel tool that could overcome the current limits of traditional approaches by collecting data in the real world and in real time. This tool was represented by a web-app for the EMA of information related to dietary habits and contextual factors that might influence dietary choices. In fact, the web-app we used—which has been previously validated against a traditional food frequency questionnaire [26]—could be useful for dietary data collection, especially among young adults who use cell phones in most aspects of daily life. Although the application of the web-app for data collection is probably the main strength of our work, some weaknesses should be discussed. Our study, in fact, included a small sample of college students from health sciences and medicine degree courses. It was a convenience sample of students who agreed to participate in the study and who used the web-app for one week. It is also worth noting that the sample was characterized by a preponderance of females. This selection bias limits the results of the study to college students who typically have good incomes, comfortable households, and structured families and who may have a greater understanding of the benefits and risks of eating well. Further research is therefore necessary to extend the results on the wider and more heterogeneous population of young adults. Moreover, we did not consider the potential effects of additional factors that were not assessed through the web-app. As an example, the web-app did not collect information on other activities during the meal period (i.e., working, studying, watching tv, listening music, reading, etc.). Along with these, additional aspects including campus lifestyle, university canteen and food costs could influence eating habits and dietary intake. Finally, it should be considered that our study was conducted during the COVID-19 pandemic, which may affect the dietary choices of human populations [31].

In conclusion, our study suggested how meal location and social context have a significant effect on the dietary choices of young adults, pointing to the need to incorporate these aspects into further epidemiological studies. In fact, identifying the additional determinants associated with healthy food choices and lifestyle factors could be crucial to design novel public health interventions targeted to adolescents and young adults. The eating environment may vary considerably between individuals, making it necessary to develop strategies that encourage healthy eating by addressing both individual and contextual factors.

## Figures and Tables

**Table 1:** Logistic regression analyses of the effect of eating episode environment on the consumption of specific foods. Logistic regression models were adjusted for age, gender, day of the week, meal type, and time of the day in which eating episodes occurred.

Foods	Characteristics of Eating Episodes	OR	95%CI	*p*-Value
Vegetables	Eating away from home vs. at home	0.52	0.36–0.76	0.001
	Eating with company vs. alone	3.09	2.31–4.13	<0.001
Fruits	Eating away from home vs. at home	0.28	0.16–0.51	<0.001
	Eating with company vs. alone	0.88	0.68–1.13	0.308
Red meat	Eating away from home vs. at home	0.83	0.51–1.35	0.455
	Eating with company vs. alone	4.39	2.51–7.65	<0.001
Processed meat	Eating away from home vs. at home	1.81	1.12–2.92	0.015
	Eating with company vs. alone	1.13	0.73–1.75	0.594
Fish	Eating away from home vs. at home	1.03	0.61–1.75	0.906
	Eating with company vs. alone	9.34	4.22–20.65	<0.001
Wholemeal bread	Eating away from home vs. at home	0.43	0.17–1.08	0.073
	Eating with company vs. alone	0.90	0.56–1.44	0.660
Nuts	Eating away from home vs. at home	0.22	0.03–1.58	0.131
	Eating with company vs. alone	0.27	0.15–0.48	<0.001
Legumes	Eating away from home vs. at home	0.13	0.01–0.95	0.044
	Eating with company vs. alone	3.03	1.56–5.88	0.001
Milk	Eating away from home vs. at home	0.53	0.24–1.18	0.122
	Eating with company vs. alone	1.51	1.11–2.06	0.009
Salty snacks	Eating away from home vs. at home	4.25	2.17–8.31	<0.001
	Eating with company vs. alone	1.63	0.69–3.85	0.265
Sugar-sweetened beverages	Eating away from home vs. at home	1.63	0.88–3.03	0.119
	Eating with company vs. alone	3.55	1.58–7.99	0.002
Alcoholic drinks	Eating away from home vs. at home	18.42	7.04–48.21	<0.001
	Eating with company vs. alone	3.98	0.52–30.31	0.183

**Table 2:** Characteristics of clusters of eating episodes. This table shows the distribution of characteristics of eating episodes according to the cluster solution obtained.

Characteristics	Cluster 1	Cluster 2	Cluster 3	Cluster 4	*p*-Value
Meal type					
Breakfast	11.1%	13.7%	45.0%	32.8%	<0.001
Morning snack	7.2%	3.5%	19.5%	3.8%	
Lunch	28.7%	38.6%	10.6%	32.2%	
Afternoon snack	8.6%	5.6%	18.8%	5.9%	
Dinner	44.4%	38.6%	6.2%	25.3%	
**Day of the week**					
Workweek	49.1%	100.0%	100.0%	0.0%	<0.001
Weekend	50.9%	0.0%	0.0%	100.0%	
**Meal location**					
At home	24.0%	100.0%	100.0%	100.0%	<0.001
At work/university	15.8%	0.0%	0.0%	0.0%	
Public closed space	16.8%	0.0%	0.0%	0.0%	
Public open space	43.4%	0.0%	0.0%	0.0%	
**Social context**					
Alone	7.5%	0.0%	100.0%	31.3%	<0.001
With family	24.7%	100.0%	0.0%	68.7%	
With friends	56.3%	0.0%	0.0%	0.0%	
With colleagues	11.5%	0.0%	0.0%	0.0%	

## Data Availability

The data presented in this study are available on request from the corresponding author.
